# PBDEs: Sjödin’s Response

**Published:** 2004-12

**Authors:** Andreas Sjödin

**Affiliations:** Center for Disease Control and Prevention, Atlanta, Georgia, E-mail: asjodin@cdc.gov

I appreciate Cleet’s response to our paper concerning time trends of polybrominated diphenyl ethers (PBDEs) and related compounds in the U.S. population (Sjödin et al. 2004), and I appreciate the opportunity to address his comments.

Pentabromodiphenyl ether (pentaBDE), along with the lower brominated congeners, was the topic of our investigation (Sjödin et al. (2004). Cleet’s statement emphasizing that the current production of PBDEs is solely in the form of decabromodiphenyl ether belittles the fact that, in 2001, 95% of the 7,500 metric tons of pentaBDE was produced and consumed in the United States [Bromine Science and Environmental Forum (BSEF) 2003]. The industry’s withdrawal of pentaBDE and octabromodiphenyl ether (octaBDE) from the market by the end of 2004 will decrease environmental output. However, continued monitoring of environmental and human levels is needed to measure exposures originating from pentaBDE and octaBDE manufactured before 2005 and to study potential exposure to decaBDE, which will continue to be manufactured.

Cleet’s second remark proposes the possibility that current human PBDE levels have reached a plateau. Because of the variability in our data (Sjödin et al. (2004) and the regionalized sampling, we believe such a conclusion may be premature. As Cleet mentions later in his letter, these studies may not be representative of U.S. and European populations. We did not claim that the sampled pools are representative. To further confirm and track our preliminary observations of human exposure to PBDEs, broader representative studies have been proposed.

Cleet’s third issue concerns comparability of our data on BDE-47 with earlier studies. We referenced several publications regarding the similarity of our measured levels to earlier findings. In a 1988 Illinois study, human levels of BDE-47 were reported to be 0.63 ng/g lipid, with a range of < 0.4–24 ng/g lipid (Sjödin et al. 2001). These Illinois levels can be contrasted to the data from serum pools collected in the southeastern United States, where we found a range of < 1–6 ng/g lipid for the same year [see Figure 1 in our paper (Sjödin et al. 2004)]. We also compared our BDE-47 levels to those in other studies: for example, 33 ng/g lipid in breast adipose tissue (range 7–200 ng/g) collected in the late 1990s (She et al. 2002); 83 ng/g lipid in a milk pool (*n* = 19) collected in 1997 in New York (Betts 2002); 130 ng/g lipid in a milk pool collected in 2000 in Austin, Texas, and Denver, Colorado (Päpke et al. 2001); and 41 ng/g lipid in milk collected in 2001 in Texas (Schecter et al. 2003). These authors reported concentrations in the same range as our study [e.g., Figure 1 in our paper (Sjödin et al. 2004)].

I appreciate Cleet’s clarification concerning production stoppage of hexabromobiphenyl (hexaBB) in Europe. Also, Cleet’s speculation about the differences in outcomes in animal studies is potentially useful. Although we did not study toxic effects of PBDEs, we asserted the cited studies to be examples of potential concern. We selected the work of Eriksson and colleagues in this regard, demonstrating observed effects in four publications: Eriksson et al. (2001, 2002), Viberg et al. (2002), and Sand et al. (2004).
